# In vitro invasion inhibition assay using antibodies against *Plasmodium knowlesi* Duffy binding protein alpha and apical membrane antigen protein 1 in human erythrocyte-adapted *P. knowlesi* A1-H.1 strain

**DOI:** 10.1186/s12936-018-2420-4

**Published:** 2018-07-27

**Authors:** Fauzi Muh, Seong-Kyun Lee, Mohammad Rafiul Hoque, Jin-Hee Han, Ji-Hoon Park, Egy Rahman Firdaus, Robert W. Moon, Yee Ling Lau, Eun-Taek Han

**Affiliations:** 1 Department of Medical Environmental Biology and Tropical Medicine, School of Medicine, Kangwon National University, Chuncheon, Gangwon-do 24341 Republic of Korea; 2 Department of Immunology and Infection, Faculty of Infectious and Tropical Diseases, London School of Hygiene and Tropical Medicine, London, WC1E 7HT UK; 3 Department of Parasitology, Faculty of Medicine, University of Malaya, Kuala Lumpur, Malaysia

**Keywords:** *Plasmodium knowlesi*, Zoonotic malaria, PkDBPα, PkAMA1, Invasion, Inhibition

## Abstract

**Background:**

The rapid process of malaria erythrocyte invasion involves ligand–receptor interactions. Inducing antibodies against specific ligands or receptors that abrogate the invasion process is a key challenge for blood stage vaccine development. However, few candidates were reported and remain to be validated for the discovery of new vaccine candidates in *Plasmodium knowlesi*.

**Methods:**

In order to investigate the efficacy of pre-clinical vaccine candidates in *P. knowlesi*-infected human cases, this study describes an in vitro invasion inhibition assay, using a *P. knowlesi* strain adapted to in vitro growth in human erythrocytes, PkA1-H.1. Recombinant proteins of *P. knowlesi* Duffy binding protein alpha (PkDBPα) and apical membrane antigen 1 (PkAMA1) were produced in *Escherichia coli* system and rabbit antibodies were generated from immune animals.

**Results:**

PkDBPα and PkAMA1 recombinant proteins were expressed as insoluble and produced as a functional refolded form for this study. Antibodies against PkDBPα and PkAMA1 specifically recognized recombinant proteins and native parasite proteins in schizont-stage parasites on the merozoite organelles. Single and combination of anti-PkDBPα and anti-PkAMA1 antibodies elicited strong growth inhibitory effects on the parasite in concentration-dependent manner. Meanwhile, IgG prevalence of PkDBPα and PkAMA1 were observed in 13.0 and 46.7% in human clinical patients, respectively.

**Conclusion:**

These data provide support for the validation of in vitro growth inhibition assay using antibodies of DBPα and AMA1 in human-adapted *P. knowlesi* parasite PkA1-H.1 strain.

**Electronic supplementary material:**

The online version of this article (10.1186/s12936-018-2420-4) contains supplementary material, which is available to authorized users.

## Background

*Plasmodium knowlesi* cases have now been reported in most countries in Southeast Asia with an epicenter of cases in Malaysia [1–3]. Until recently *P. knowlesi* cases were commonly misdiagnosed as *P. malariae* due to its similar morphology and less sensitive diagnostic tests, masking the extent of infections in the region [2, 4, 5]. *Plasmodium knowlesi* can cause severe disease in humans and life-threatening malaria cases have been reported [6]. Despite this, research into this parasite has been relatively neglected compared to other human malaria infections. The recent adaptation of *P. knowlesi* to human erythrocyte in vitro culture has provided an important new tool to study parasite biology as well as the opportunity for new assays to test therapeutic interventions against this important parasite.

The *Plasmodium* life cycle is complex and involves ligand–receptor interactions for successful invasion and multiplication in host erythrocytes [7]. However, *Plasmodium vivax* and *P. knowlesi* depend on the presence of the Duffy antigen receptor for chemokine (DARC) on erythrocytes for invasion. *P. knowlesi* Duffy binding protein alpha (PkDBPα) is a ligand for DARC and pivotal in human infection [8, 9]. Antibodies against domain II of PkDBPα or against surface-exposed DARC epitope inhibited invasion of monkey and human erythrocytes [10]. While apical membrane antigen 1 (AMA1) is one of the polymorphic leading vaccine candidates, which involved in tight junction formation and essential for an invasion among *Plasmodium* species [11–14]. Anti-PkAMA1 antibody inhibited erythrocyte invasion in an animal model [15]. The complex formed with the ectodomain of PkAMA1 (domain 1 and 2) and rhoptry neck protein 2 (RON2) is essential for invasion and as compared to *Plasmodium falciparum* and *P. vivax,* the RON2-binding site of PkAMA1 is much less polymorphic [13].

Few vaccine candidates directed to blood stage antigens have been only evaluated to inhibit *P. knowlesi* invasion into either monkey or human erythrocytes [16], such as PkDBPα [10] and PkAMA1 [14]. Antibodies play a major role in immunity to malaria by inhibiting the invasion into host red blood cells. Antibodies that abrogate invasion are extensively studied in *P. falciparum* and an association with protection against malaria could be shown [17, 18]. In *P. vivax,* the functional assessment of inhibitory antibodies is less well studied due to the lack of a continuous in vitro culture [19].

Invasion inhibition assays or growth inhibition assays have been designed to evaluate malaria vaccine candidates for *P. falciparum* and *P. vivax* [20–22]. A reliable, sensitive and validated invasion inhibition assay is required for evaluation of protective antibodies to malaria [17]. Several methods were used to determine parasitaemia for in vitro growth inhibition assays, such as counting parasitaemia by Giemsa-staining and microscopy [23, 24], or measuring parasite lactate dehydrogenase (pLDH) activity [17, 25], but both have low sensitivity in the assessment of parasite invasion. Flow cytometry is a reliable and versatile technique and widely used because it is highly reproducible, efficient, and more robust than other methods [17, 26, 27]. Previous studies had shown the inhibitory effect of anti-PkDBPα and anti-PkAMA1 antibodies with different assays, such as for the presence of rings on Giemsa-stained smears and pLDH activity, respectively [10, 14]. To assess the potential of new vaccine candidates against the blood stages of *P. knowlesi*, a high throughput functional screening assay is required.

In this study, PkDBPα and PkAMA1 antigens were successfully produced as correctly folded forms in *Escherichia coli* system which is different from previous studies [10, 14] and antibodies raised from immune animals were validated in recognition of native parasite antigen with subcellular localization in blood-stage parasites. IgG prevalence was measured with knowlesi human clinical patients. Previous studies had described the invasion-inhibitory activity of PkDBPα and PkAMA1 antibodies in *P. knowlesi* H strain with Giemsa-stained smears in presence of rings or parasite-specific lactate dehydrogenase (pLDH) activity assay [10, 14]. In this study, a flow cytometric based invasion inhibition assay was used to validate inhibitory antibodies to abrogate *P. knowlesi* invasion into human erythrocytes with human-adapted *P. knowlesi* PkA1-H.1 strain parasites and it may be useful for discovery of new antigenic and vaccine candidates in the future.

## Methods

### Patients’ sample information

Blood samples of clinical cases were collected in EDTA-vacutainer from University of Malaya Medical Center (UMMC), Malaysia between 2010 and 2013 at day 0 (15 patients; mean age 46 years, range 22–70 years; mean parasitaemia 0.18, range 0.03–0.30) [28]. Samples were centrifuged and the serum was separated. All *P. knowlesi* chosen for this study have been confirmed by microscopy and species-specific PCR as described elsewhere [5]. Serum was also collected from healthy individuals living in non-endemic areas of the Republic of Korea (ROK); these samples were used as a negative control. All experiments were performed in accordance with relevant guidelines and regulations and all experimental protocols involving human samples approved by the Kangwon National University Hospital Ethical Committee (IRB No. 2014-08-008-002) and the University of Malaya Medical Ethics Committee (Ref No. 817.18) and the Medical Research Ethic Committee (MREC), Ministry of Health, Malaysia (National Medical Research Register ID No. 13079). Informed consent was obtained from all subjects.

### Parasite culture

Human-adapted *P. knowlesi* A1-H.1 strain was maintained with fresh human erythrocytes in RPMI-1640-based medium (Invitrogen Life Technologies, Grand Island, NY) containing other components in addition of human AB serum as described previously [29].

### Recombinant protein expression

The DNA fragment of PkDBPα-II was amplified from the *P. knowlesi* A1-H.1 strain with specific primers PkDBPα_F (actgat*ccatgg*TTATTAATCAAACTTTTCTTC); PkDBPα_R (atagttta*gcggccgc*TCAGAGATGATGATGATGATGTTCAGTTATCGGATTAA) as described previously [30]. Restriction enzymes, *Nco*I and *Not*I, were indicated as italicized and underlined letters. PkAMA1 domain I-II was amplified with specific primers; PkAMA1_F (ggtcgcggatcc*gaattc*ATGCCTATCATTGAGAGAAGTATACG) and PkAMA1_R (ggtggtggtg*ctcgag*TGGAAATTCATTGTCTACTTCTTGAG). Restriction enzymes, *Xho*I and *Bam*HI, were indicated as italicized and underlined letters. PCR was run using a high-fidelity KDO-plus Kit (Toyobo Co., Osaka, Japan) with initial denaturation at 94 °C for 2 min, followed by 35 cycles of 94 °C for 15 s, 60 °C for 30 s, and 58 °C for 1.5 min and a final extension at 68 °C for 10 min. The amplicons were purified using a gel extraction kit and ligated into the pET28a(+) expression vector (Novagen, Madison, WI) with His-tag. The obtained plasmid was confirmed by DNA sequencing analysis and transformed into *E. coli* Origami™ 2(DE3) pLySs (Novagen) and BL21 (DE3) competent cells (Novagen) for PkDBPα and PkAMA1, respectively. Recombinant proteins were induced with 0.1 mM isopropyl-β-d-thiogalactopyranoside (IPTG; Sigma-Aldrich Co., St. Louis, MO). Protein solubilization, purification and refolding were described elsewhere [31]. The refolded proteins were eluted from ion exchange chromatography (HiTrap™ SP FF; GE Healthcare Life Sciences) using gradient 0.2 and 1 M NaCl.

### SDS-PAGE and western blot analysis

Refolded proteins of DBPα-II and AMA1_DI-II of *P. knowlesi* were separated by 10% SDS-PAGE under reducing and non-reducing conditions and stained with 0.25% Coomassie brilliant blue R-250 (Sigma-Aldrich Co.). Bovine serum albumin (BSA, 5 µg; Sigma-Aldrich Co.) was used as positive control. Briefly, 2.5 µg refolded proteins and 5 µg bovine serum albumin (BSA) protein were incubated with 10 mM dithiothreitol (DTT, reduced condition), incubated at 37 °C for 1 h and added 2× loading dye containing reducing agent 2-mercaptoethanol; 2.5 µg proteins and 5 µg BSA protein without DTT (non-reduced condition), added 2× loading dye without reducing agent. Samples were heated at 95 °C for 4 min.

The parasite lysate was prepared as described previously [28]. Parasite lysate and recombinant proteins (0.5 μg) were separated by 13% SDS-PAGE under reducing condition. Membranes were transferred to a 0.45-µm polyvinylidene fluoride (PVDF) membrane (Millipore, Billerica, MA) in a semi-dry transfer apparatus (ATTO corp., Japan) at a current of 400 mA for 40 min. Membrane-transferred proteins were reacted with primary anti-His (1:2000) monoclonal antibody, anti-rabbit antibodies (1:2000, recombinant protein recognition or 1:50, parasite lysate recognition) and then reacted with secondary IRDye goat anti-mouse or goat anti-rabbit antibodies (1:10,000) (LI-COR Bioscience, Lincoln, NE). The Odyssey infrared imaging system (LI-COR Bioscience) and Odyssey software (LI-COR Bioscience) were used to visualize the bands.

### Antibody production and IgG purification

Immunization was conducted in Japanese white rabbit with 250 μg of folded recombinant proteins in Freund’s complete adjuvant followed by Freund’s incomplete adjuvant in the 2-week interval. Rabbits were injected for 3 times and serum samples were collected 2 weeks after final boost. Total IgGs were purified from 1 mL of rabbit serum by using protein G HP column according to the manufacturer’s protocol (GE Healthcare Life Sciences). The eluted IgGs fractions were dialyzed against incomplete RPMI1640 (Invitrogen Life Technologies) and concentrated with centrifugal devices 30 kDa cut-off value (Millipore, Billerica, MA). To remove any nonspecific inhibitory effect of IgGs, the purified IgGs were preadsorbed with human erythrocytes as described previously [22] with little modification (25 µL of erythrocytes per 4 mg IgGs) for 30 min.

### Immunofluorescence assay (IFA)

Schizont parasites of *P. knowlesi* were fixed with ice-cold acetone. Anti-PkDBPα and PkAMA1 (1:100) polyclonal serum were reacted as primary antibody and Alexa Fluor 488-conjugated anti-rabbit (H + L) antibody (1:500, Invitrogen Life Technologies) was used as secondary antibody. The slides were visualized using a confocal laser scanning microscope (FV200 Olympus, Tokyo, Japan) equipped with 20× dry and 60× oil objectives. Images were captured using the FV10-ASW 3.0 viewer software.

### Protein microarray

Amine-coated slides were used and spotted in duplicate with 100 ng of purified recombinant proteins of PkDBPα or PkAMA1 at 37 °C for 2 h. After blocking with 5% BSA in PBS 0.1% Tween-20, knowlesi-infected patients’ serum and healthy individuals’ serum were reacted (1:25) for 1 h. Alexa Fluor 546-conjugated goat anti-human IgG (10 µg/mL; Invitrogen Life Technologies) was used as a secondary antibody, and the fluorescence signal was detected with a scanner (InnoScan scanner, Carbonne, France). The cut-off values were set to the mean fluorescence intensity (MFI) plus 2 standard deviations (SDs). The normalized MFI was calculated by dividing the MFI by the cut-off value.

### Growth inhibition assay (GIA)

*Plasmodium knowlesi* A1-H.1 schizonts were purified by using magnetic-activated cell sorting (MACS) technology (Miltenyi Biotec, Bergisch Gladbach, Germany) with an LD column (Miltenyi Biotec) and cultured in 96-well plates in a total 100-µL volume in each well. Hematocrit and initial parasitaemia were adjusted to 2 and 1.5%, respectively. Purified anti-PkDBPα or PkAMA1 rabbit IgGs at different concentrations (0.5, 1.0, 1.5, and 2.0 mg/mL) were added to a 96-well plate (test wells) during *P. knowlesi* sub-culture. Combination of anti-PkDBPα and PkAMA1 IgGs was added to make final 0.5 (0.25 mg/mL each), 1.0, 1.5 and 2.0 mg/mL per well. Control wells without antibody (normal invasion well) were set to validate the normal invasion ability of the parasite. The culture was incubated at 37 °C in a humidified culture chamber for approximately 10 h until newly invaded ring-stage parasites were found. The non-immunized rabbit IgG and anti-Fy6 monoclonal antibody against DARC (2C3; 25 μg/mL; a kind gift from Renia L, Singapore Immunology Network-BMSI-A STAR) [32] were used as a baseline control. This assay was performed independently in duplicate twice.

### Determination of parasitaemia by flow cytometry and microscopy

Parasites were stained with SYBR Green I (Sigma-Aldrich Co.). Briefly, cultures were centrifuged at 500×*g* for 5 min and pellets were washed two times with filtered 1× PBS and fixed with 0.05% glutaraldehyde (Sigma-Aldrich Co.) for 10 min. Fixed samples were washed twice, stained with SYBR Green I at 0.2× dilution for 10 min, and washed twice with PBS. The samples were analyzed with an Accuri C6 flow cytometer (Accuri cytometers Inc., Ann Arbor, MI). In total, 200,000 cells were recorded. Percent inhibition was calculated using the following formula: 100 − (100 × (test well (P3)/normal invasion well (P3)). After 10 h post-invasion, thin smears were made and stained with 10% Giemsa (Merck, Germany) for 10 min. The infected erythrocytes and the morphology of rings were counted and confirmed under a light microscope. More than 20,000 erythrocytes were counted. Percent inhibition was calculated using the same formula as for flow cytometry. Two expert persons in the lab were recruited in the counting of parasitaemia by microscopy to maintain the objectivity and the persons were blinded by the information of each smear.

### Statistical analysis

All calculations and data analysis were performed with GraphPad PRISM 5 (GraphPad Software, Inc., CA, San Diego, CA, USA). The independent non-parametric Mann–Whitney test was used to determined differences between means with 95% CI. Value of *p *< 0.05 was considered statistically different. The level of agreement of two measuring methods, flow cytometry and microscopy, was validated using the Bland–Altman plot [33, 34].

## Results

### Recombinant protein expression of PkDBPα and PkAMA1

**Fig. 1:**
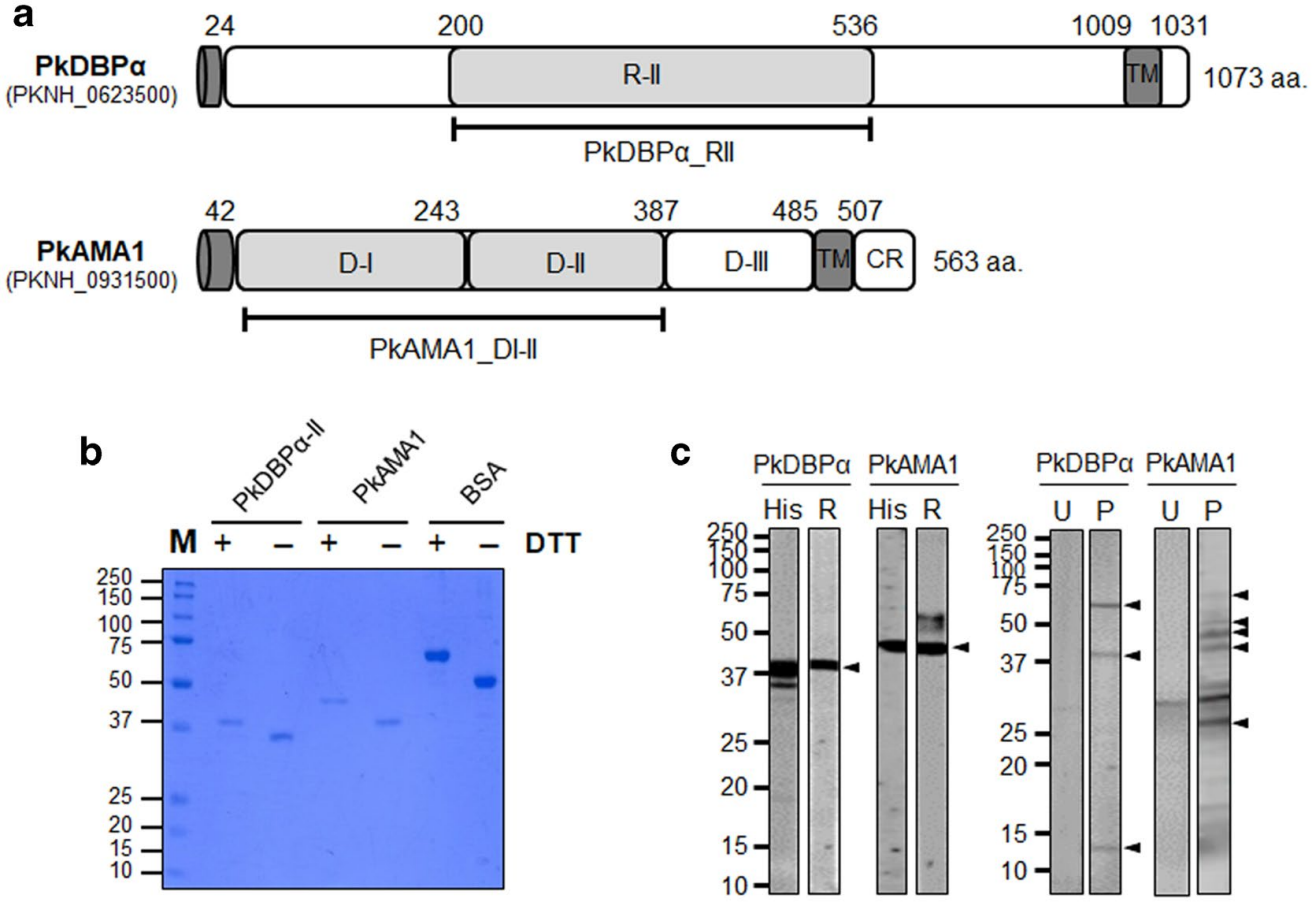
Schematic structure of PkDBPα and PkAMA1 protein expression. **a** Schematic of PkDBPα region II (200–536 aa) and PkAMA1 domain I and II (43–387 aa). **b** Reduced (+) and non-reduced (−) condition of refolded-PkDBPα and PkAMA1 (2.5 and 5 µg for BSA were loaded per well). **c** Western blot analysis of recombinant proteins PkDBPα and PkAMA1, and PkA1-H.1 parasite lysate probed with antibodies. *His* anti-His antibody, *R* rabbit antibodies, *U* uninfected RBCs, *P* parasite lysate. Black head arrows, specific fragmentation bands

Purified recombinant protein of PkDBPα region II (200–536 aa.) and PkAMA1 domain I–II (43–387 aa.) have been successfully produced and refolded in this study and used for rabbit antibody production (Fig. 1a). The bands migrated in different mobility between reduced (DTT +) and non-reduced condition (DTT −) on the SDS-PAGE (Fig. 1b). Anti-PkDBPα, anti-PkAMA1 and anti-His antibodies recognized the recombinant proteins and parasite lysate by western blot analysis (Fig. 1c). These results suggest that the native condition of refolded proteins had been formed and its antibody can recognize the recombinant protein and native parasite protein.

### Recognition of native PkDBPα and PkAMA1 proteins from cultured parasites

**Fig. 2:**
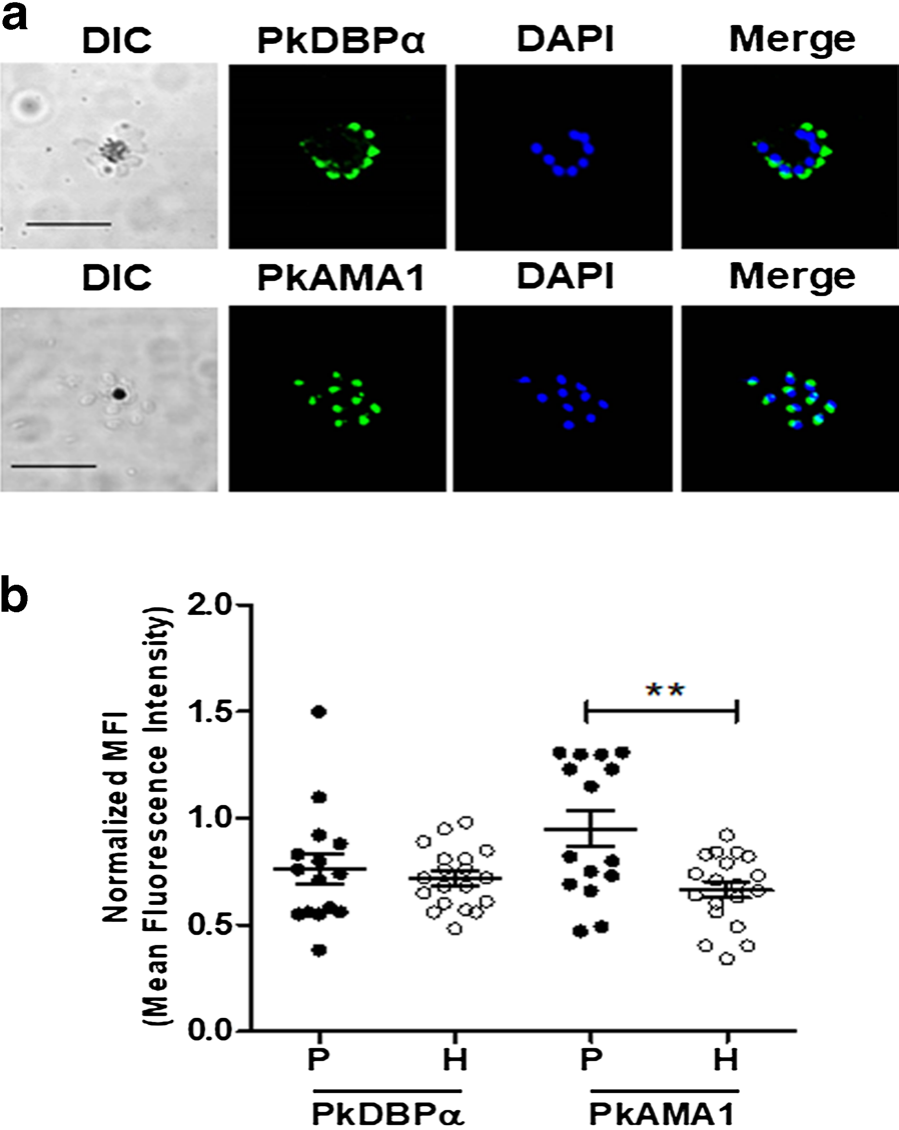
Recognition of PkDBPα and PkAMA1 protein in the parasite and human patients. **a** Immunofluorescence assay of anti-PkDBPα and anti-PkAMA1 antibodies with PkA1-H.1 schizont. DAPI, 4′,6-diaminidino-2-phenylindole. Bars indicate 5 μm. **b** The humoral immune response of PkDBPα and PkAMA1. The IgG response is represented by normalized mean fluorescence intensity [MFI]: MFI of the test sample/[MFI + 2 standard deviations]. *P* patient serum, *H* healthy individual serum. Black circle, individual patient serum; white circle, healthy individual serum; double asterisks, *p *< 0.001

To validate antibody against those targets, immunofluorescence revealed that those antibodies specifically recognized schizont stage of *P. knowlesi* on apical end organelles (Fig. 2a). Human knowlesi-infected patients’ serum also showed higher reactivity than that healthy individual serum samples for PkAMA1 but not to PkDBPα (Fig. 2b; Table 1). These data suggest that refolded proteins and antibodies were successfully produced in this study.

**Table 1:** IgG responses of knowlesi-infected patients’ serum samples to PkDBPα and PkAMA1 recombinant proteins

Samples	No. of patient samples (*n*)			Normalized MFI^e^	No. of healthy samples (*n*)			Normalized MFI^e^
	Positive^a^	Negative^b^	Total (%)^c^		Positive^a^	Negative^b^	Total (%)^d^	
PkDBPα	2	13	15 (13.0)	0.76	0	19	19 (100.0)	0.72
PkAMA1	7	8	15 (46.7)	0.92	0	19	19 (100.0)	0.67

^a^Positive: the individual who is reactive to its particular antigen

^b^Negative: the individual who is not reactive to its particular antigen

^c^Sensitivity: percentage of positive-malaria patient samples

^d^Specificity: percentage of negative-healthy individual samples

^e^Normalized MFI: mean fluorescence intensities were divided by a cut-off value + 2 standard deviations above the mean fluorescence intensity of the malaria-naïve samples

### Ex vivo invasion assay

**Fig. 3:**
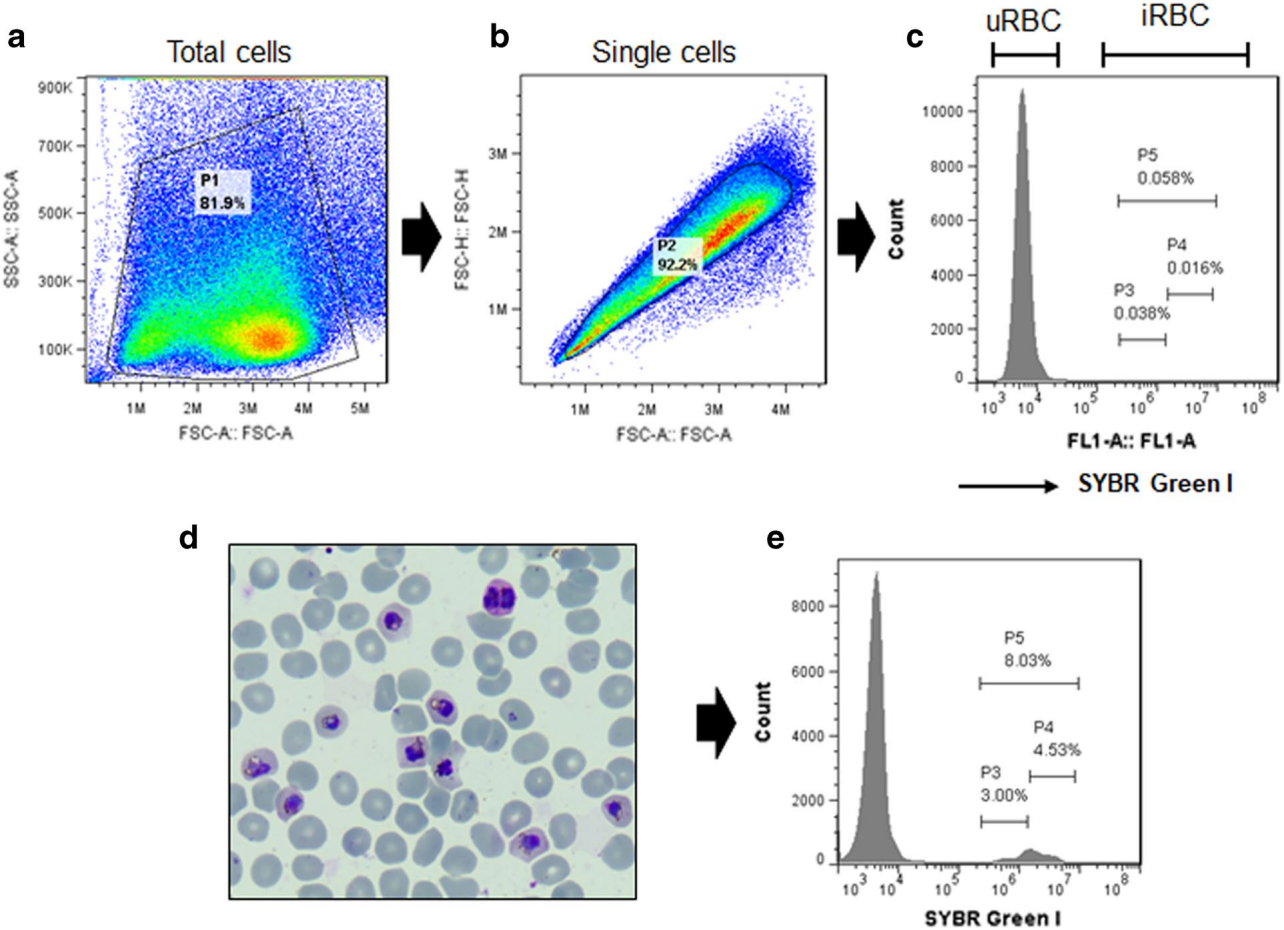
Gating strategy. **a** Total cell gating strategy from uninfected erythrocytes with forward scatter-area (FSC-A) and side scatter-area (SSC-A). **b** Single cell gating from uninfected erythrocytes with forward scatter (FSC-A and FSC-H). **c** Histogram flow cytometry of uninfected erythrocytes with X-axis FL1-A (SYBR Green I). **d** Giemsa staining of in vitro culture of *P. knowlesi.* **e** Histogram flow cytometry of *P. knowlesi* invasion. P1, total cells of being counted; P2, single cells of being counted; P3, early rings; P4, late trophozoites and schizonts; P5, infected erythrocytes (P3 + P4)

The flow cytometry assay using SYBR Green I dye was performed 10 h post-invasion. Total cells were gated from uninfected erythrocytes on the basis of forward scatter-area (FSC-A) and side scatter-area (SSC-A) (Fig. 3a). Then, FSC-A and FSC-H were used to get single cells (Fig. 3b). Infected and uninfected erythrocytes were adjusted by FL-1 (FITC) histogram (Fig. 3c). This gating strategy clearly distinguished between newly invaded rings (P3) and late trophozoites and schizonts (P4), while P5 was counted as total parasitaemia (P3 + P4) (Fig. 3d, e).

**Fig. 4:**
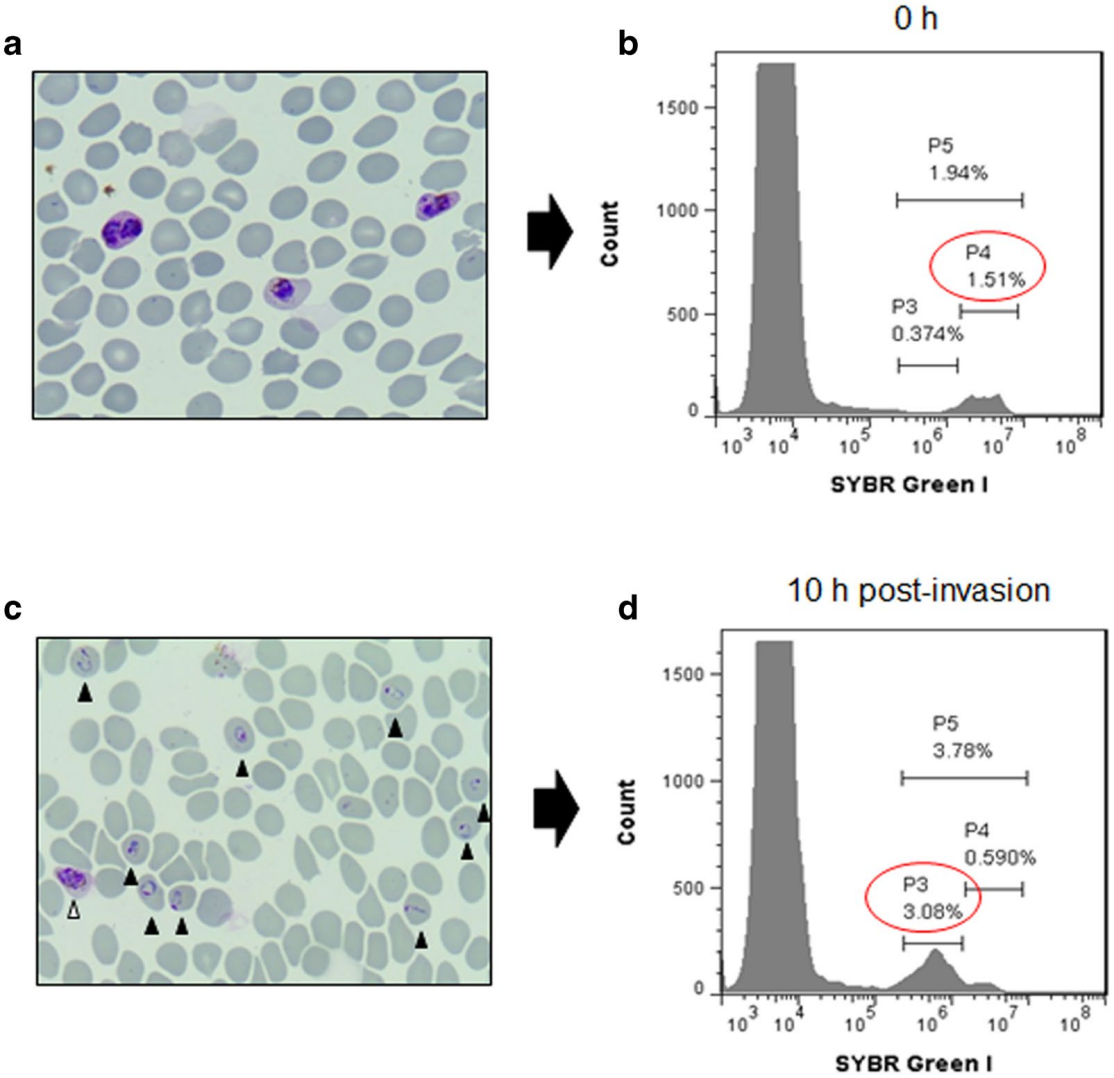
Invasion ability in 96-well plate. **a**, **c** Giemsa staining of *P. knowlesi* before (0 h) and after the invasion (10 h). **b**, **d** Histogram flow cytometry of before (0 h) and post-invasion (10 h). White head arrow, schizonts; black head arrows, newly rings

To investigate the invasion ability of PkA1-H.1, schizonts were purified and transferred back to culture in the 96-well plates in a 100 μL culture volume (Fig. 4). At 0 h, schizonts were added to the well at approximately 1.5% parasitaemia counted by microscopy (Fig. 4a). The flow cytometry histogram of showed defined populations and rings (P3) and schizonts (P4) could be readily distinguished (Fig. 4b). After 10 h, newly invaded ring stage parasites were found (Fig. 4c, black head arrows), but some schizonts were found that had not ruptured yet (white head arrow). These two populations were observed by flow cytometry with P3 and P4 population ratios consistent with the majority of parasites being ring stages (Fig. 4d).

### In vitro growth inhibition assay (GIA)

**Fig. 5:**
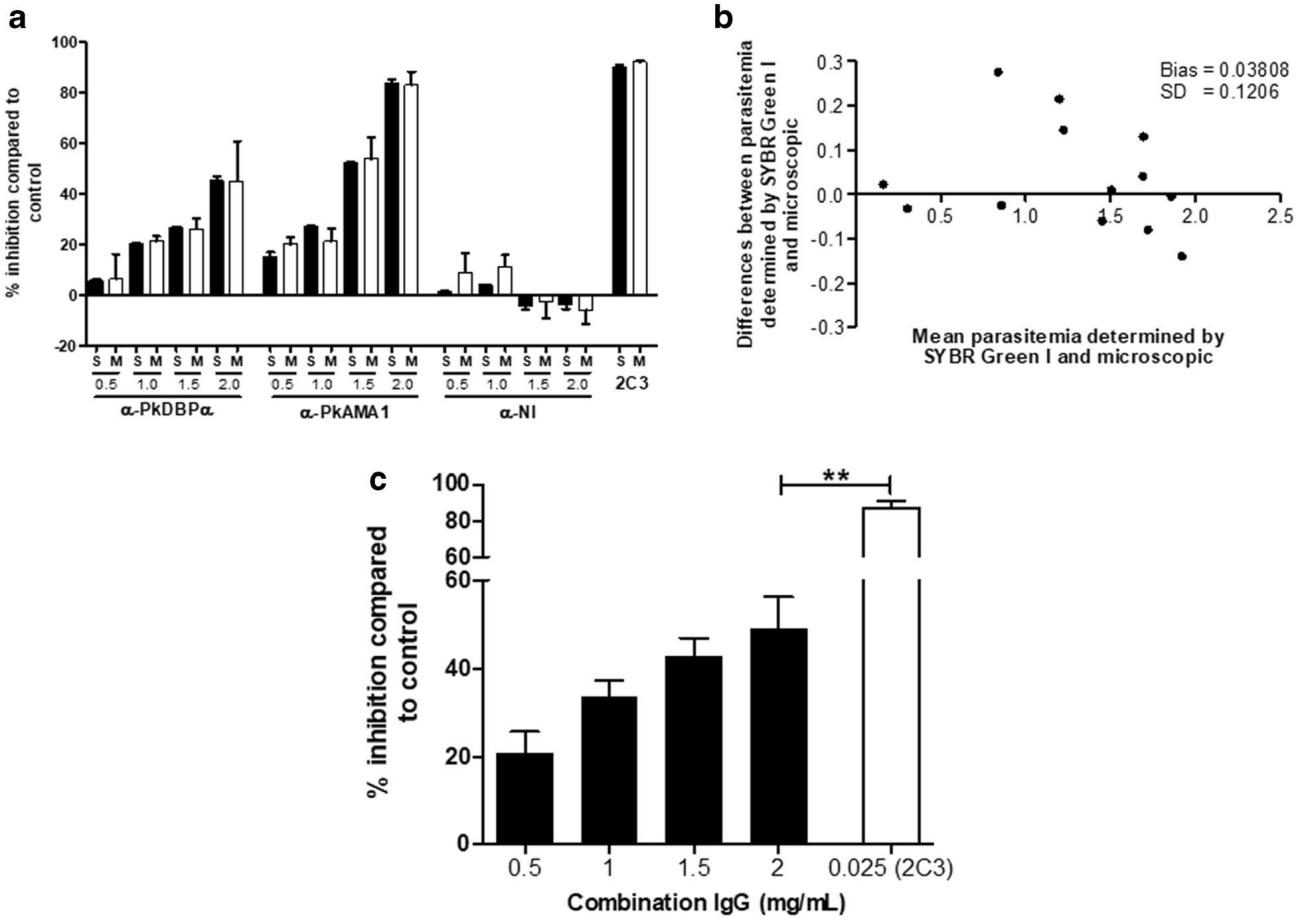
Application of invasion inhibition assay. **a** Comparison of invasion inhibition efficiency of gradient concentration of antibodies by using flow cytometry and microscopy. *S* SYBR Green I flow cytometry, *M* microscopy, *αNI* non-immunized IgG. **b** Agreement of parasitaemia determination by flow cytometry and microscopy. *SD* standard deviation. **c** Invasion-inhibition activity of antibody combination. Gradient concentration IgG of PkDBPα and PkAMA1 to make final concentration 0.5; 1.0; 1.5 and 2.0 mg/mL were added. Double asterisks, *p *< 0.001

In order to know the functional activity of antibodies to block PkA1-H.1 invasion into human erythrocytes, anti-PkDBPα and anti-PkAMA1 IgGs were added to the culture in 96-well plates in different concentrations (0.5, 1.0, 1.5 and 2.0 mg/mL). Both antibodies demonstrated high inhibitory effects on *P. knowlesi* invasion into human erythrocytes in a concentration-dependent manner in contrast to non-immunized IgG where little and no inhibition or little induce the invasion were observed (Fig. 5a). The anti-DARC monoclonal antibody (2C3, 25 µg/mL) almost completely abrogated the invasion (90.34 ± 1.22). Flow cytometry and microscopic data showed that these two methods were comparably effective at distinguishing newly invaded rings after 10 h post-invasion (Fig. 5a, b) (Additional files 1, 2). The level of agreement of two methods was showed with Bland–Altman analysis with less bias (0.03808) and standard deviation (SD, 0.1206) (Fig. 5b). It indicates that FACS data with SYBR Green I is less variable than microscopy. Additive effect of antibodies was observed using combination of anti-PkDBPα and anti-PkAMA1 in a concentration-dependent manner (Fig. 5c). This data indicates that combination of different functional antibodies gives more potent than a single antibody.

## Discussion

The emergence of zoonotic *P. knowlesi* is thought to be a new hurdle in malaria elimination programs, and human *P. knowlesi* cases are on the rise in Southeast Asian countries [3, 6, 35–37]. Therefore, effective vaccines and new therapeutic strategies are needed in these endemic countries. The rapid discovery of new vaccine candidates in *P. falciparum* and *P. vivax* necessitates a functional high-throughput assay to evaluate invasion inhibition. A growth inhibitory assay or invasion inhibition assay is required to evaluate the functional activity of vaccines in pre-clinical studies. Evaluation of invasion inhibition has been conducted by using target-specific antibodies [16, 17, 20, 27]. In this study, a high-throughput invasion inhibition assay based on a versatile, reproducible and reliable flow cytometry based-assay was used in the critical evaluation of pre-clinical vaccine candidates against *P. knowlesi*. In order to quantify newly invaded ring-stage *P. knowlesi* parasites after 10 h post-invasion, SYBR Green I was used as a nucleic acid dye for enumerating infected parasites in this study. This method was applied for evaluation of *P. knowlesi* invasion ability with normocyte in a previous study [38]. However, in this study, antibodies were used to evaluate the invasion inhibition against *P. knowlesi* specific and important parasite ligand molecules, PkDBPα and PkAMA1.

In this study, refolded proteins of PkDBPα and PkAMA1 have been successfully produced in *E. coli* system. The correctly folded proteins were confirmed by SDS-PAGE analysis as shown in different migration mobility of reduced and non-reduced proteins by DTT. The antibodies also recognized apical end organelle of merozoites and fragmentation of native protein by western blot. It supports that protein and antibody productions in this study worked well and different systems were used in previous studies [10, 14]. SYBR Green I was extensively used in the enumeration of parasitaemia because it strongly binds to double-stranded DNA and has a low binding affinity for binding single-stranded RNA [39–41]. In the 96-well plate, the *P. knowlesi* enabled to grow and rupture and one cycle of complete invasion can be achieved within 24 h [29]. By using FL-1 (FITC) flow cytometry histogram, uninfected and infected erythrocyte population could be distinguished (Fig. 3). Furthermore, to detect new invasion events, two distinct populations from infected erythrocytes were clearly defined as newly invaded rings (P3) and trophozoite/schizonts (P4) (Figs. 3, 4). The early ring stages was harvested to allow quantification of parasite invasion by differentiation in the number of nuclei and amount of nucleic acid [39, 40]. SYBR Green I is more versatile dye than others because it takes advantages of the brighter signal in a flow cytometer of infected erythrocyte counting [42, 43]. This study also reported that FACS analysis and microscopy are not different in the enumeration of parasitaemia.

The antibodies against the micronemal protein of PkDBPα-II had been reported to abrogate the *P. knowlesi* invasion into human and monkey erythrocytes [10]. Significant inhibition was also observed with polyclonal anti-PvDBP-II antibody in human clinical *P. vivax* isolates (32.7%) [19]. Likewise in *P. vivax, P. knowlesi* invasion depends on the Duffy antigen on erythrocytes. Blocking of DBPα ligand had been thought to block the interaction with DARC receptor on human erythrocytes and its interaction is essential for invasion of human erythrocytes [44–46]. Thus, human-adapted *P. knowlesi* A1-H.1 was successfully maintained in long-term in vitro culture in recent and antibody against PkDBPα-II showed reduction of invasion to human erythrocytes in concentration-dependent manner. Blocking of DARC receptor by the anti-Fy6 monoclonal antibody (2C3) almost 100% abrogated the invasion of *P. knowlesi* into human erythrocytes similar as previous reports [16, 27]. As one of the leading vaccine candidates of micronemal protein, apical merozoite membrane antigen 1 (AMA1) had been widely studied that antibodies to AMA1 effectively inhibited invasion into human erythrocytes in concentration-dependent manner [47–49]. Similarly, anti-PkAMA1 domain I–II also showed the abrogation of invasion into human erythrocytes in concentration-dependent manner, highlighting the essential role of AMA1 in complex with RON2 [14, 15]. Thus, this study has successfully confirmed the functional activity of PkDBPα and PkAMA1 proteins by *E. coli* expression system and being comparable with previous findings with different systems. Interestingly, a combination of PkDBPα and PkAMA1 antibody showed the addictive effect to inhibit the PkA1-H.1 merozoites invasion following the concentration-dependent manner. The expansion of development effective vaccine targeting combination of merozoite antigens has become a priority to be more effective to control parasites to evolve from host immune response [50]. The functional difference of PkDBPα and PkAMA1 in invasion and in a combination of its antibody could be more potent to abrogate the invasion cascade of *P. knowlesi* to invade human erythrocytes. Moreover, the anti-PkDBPα and anti-PkAMA1 antibodies were prevalent, but in this study invasion inhibition activity of human antibodies to PkDBPα and PkAMA1 was not investigated. PkDBPα seems low reactivity may be due to its high genetic diversity in region II among Malaysian clinical isolates. Thus, further study is needed to show the possible of cross-protection in human clinical patients against different strain of *P. knowlesi*.

## Conclusions

In summary, antibodies used in this study have validated in vitro invasion inhibition assay by using human-adapted *P. knowlesi* PkA1-H.1 strain parasite. Combination of antibodies with the functional difference may be useful to develop the effective vaccine against zoonotic *P. knowlesi.*

## Additional files

**Additional file 1.** FACS analysis of invasion inhibition assay. P1, total cells of being counted; P2, single cells of being counted; P3, early rings; P4, late trophozoites and schizonts; P5, infected erythrocytes (P3 + P4).

**Additional file 2.** Microscopic analysis after 10 h post-invasion. Arrows, newly rings; NI, non-immunized IgG.
