# Supplementary material for: In vitro invasion inhibition assay using antibodies against Plasmodium knowlesi Duffy binding protein alpha and apical membrane antigen protein 1 in human erythrocyte-adapted P. knowlesi A1-H.1 strain
Source: Malar J. 2018 Jul 27;17:272. doi: 10.1186/s12936-018-2420-4 (PMC6062950; doi:10.1186/s12936-018-2420-4)

Additional File 1. FACS analysis of invasion inhibition assay. P1, total cells of being counted; P2, single cells of being counted; P3, early rings; P4, late trophozoites and schizonts; P5, infected erythrocytes (P3+P4).

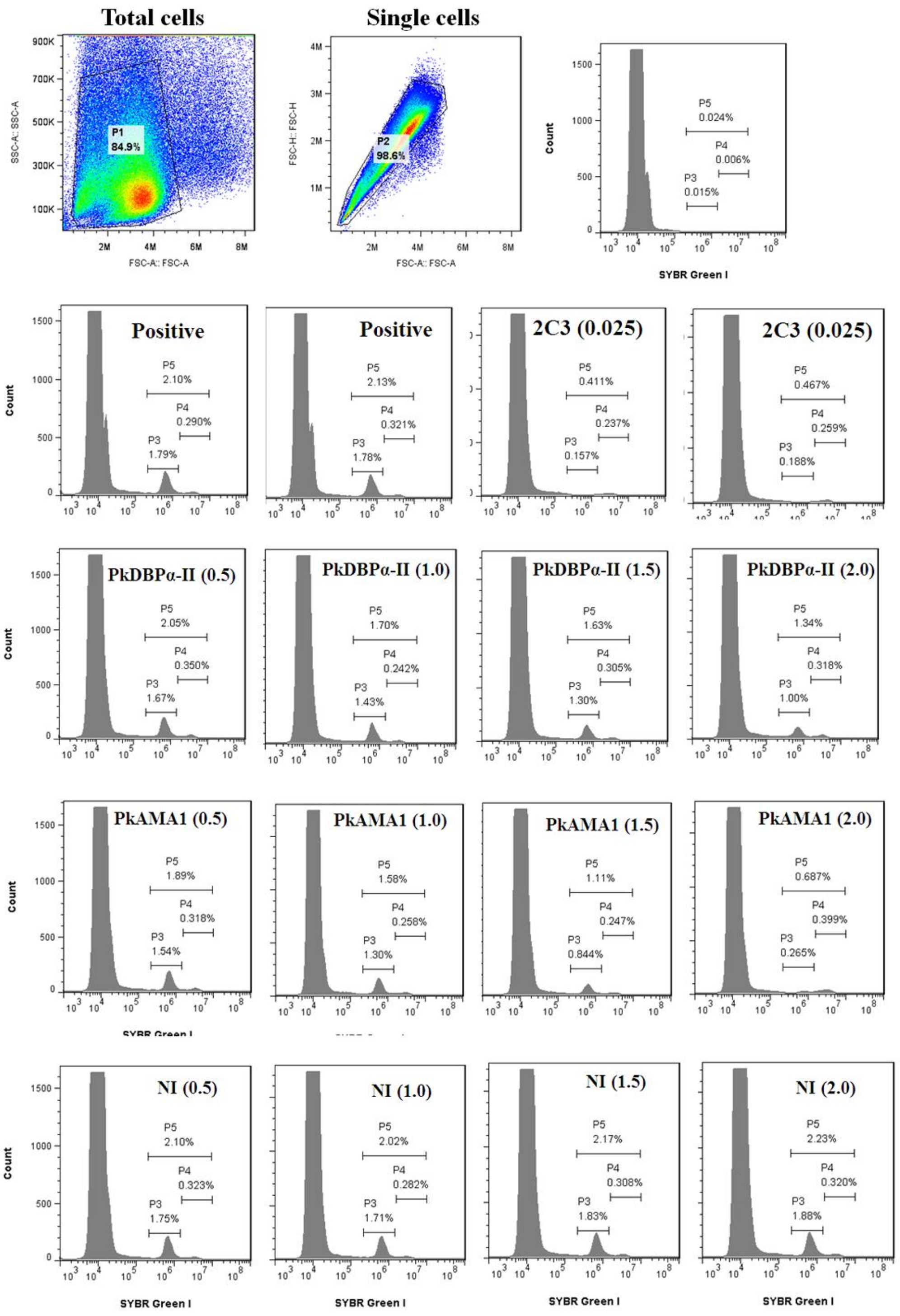

Supplement: Supplementary file 1 — Additional file 1. FACS analysis of invasion inhibition assay. P1, total cells of being counted; P2, single cells of being counted; P3, early rings; P4, late trophozoites and schizonts; P5, infected erythrocytes (P3 + P4). [file 12936_2018_2420_MOESM1_ESM.pdf]
