# Supplementary material for: In vitro invasion inhibition assay using antibodies against Plasmodium knowlesi Duffy binding protein alpha and apical membrane antigen protein 1 in human erythrocyte-adapted P. knowlesi A1-H.1 strain
Source: Malar J. 2018 Jul 27;17:272. doi: 10.1186/s12936-018-2420-4 (PMC6062950; doi:10.1186/s12936-018-2420-4)

Additional File 2. Microscopic analysis after 10 h post-invasion. Arrows, newly rings; NI, non-immunized IgG.

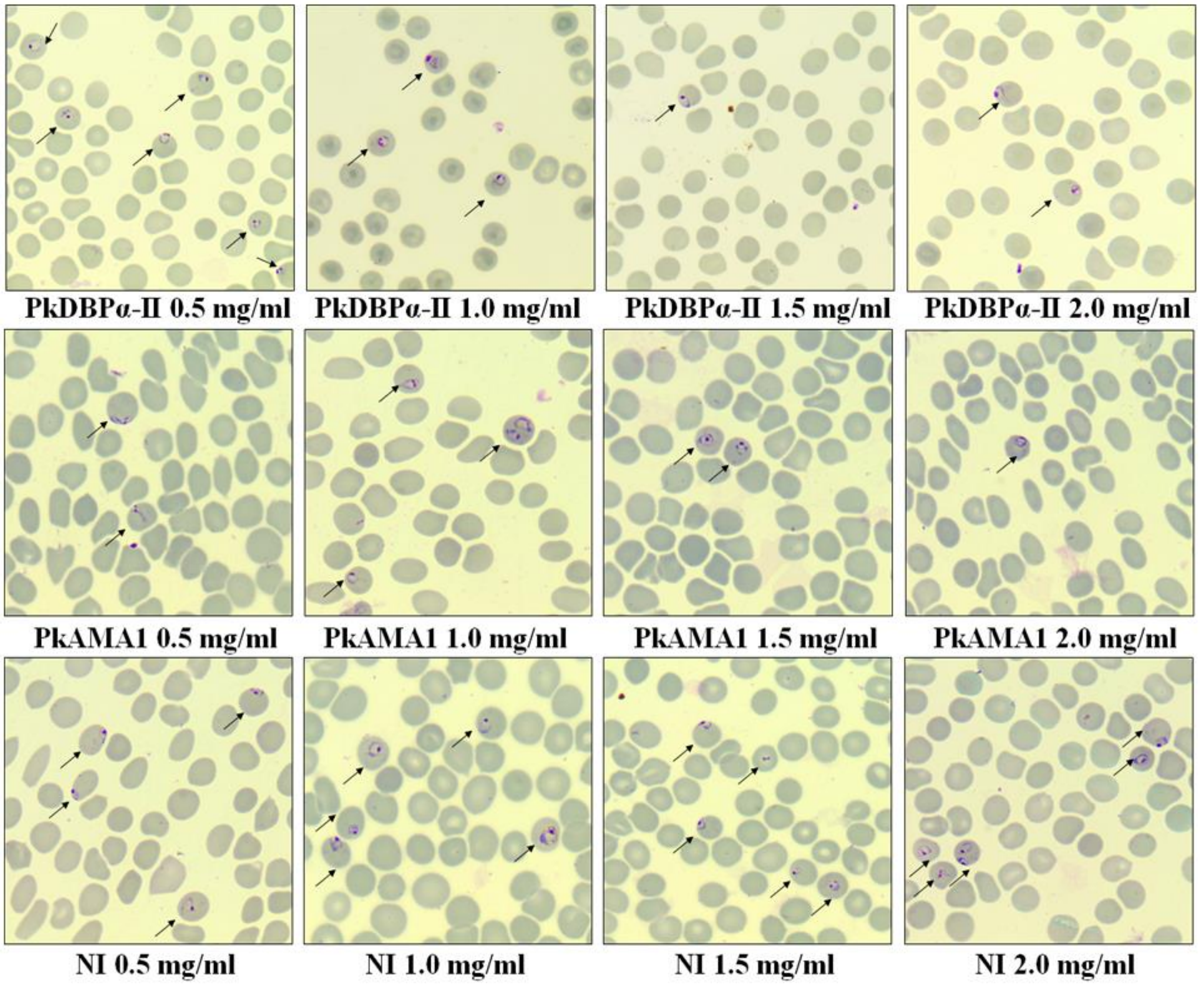

Supplement: Supplementary file 2 — Additional file 2. Microscopic analysis after 10 h post-invasion. Arrows, newly rings; NI, non-immunized IgG. [file 12936_2018_2420_MOESM2_ESM.pdf]
